# Approaches to PET Imaging of Glioblastoma

**DOI:** 10.3390/molecules25030568

**Published:** 2020-01-28

**Authors:** Lindsey R. Drake, Ansel T. Hillmer, Zhengxin Cai

**Affiliations:** 1 Yale PET Center, Yale University School of Medicine, New Haven, CT 06511, USA; ansel.hillmer@yale.edu (A.T.H.); jason.cai@yale.edu (Z.C.); 2 Department of Radiology and Bioimaging Sciences, Yale University School of Medicine, New Haven, CT 06511, USA; 3 Department of Psychiatry, Yale University School of Medicine, New Haven, CT 06511, USA; 4 Department of Biomedical Engineering, Yale School of Engineering and Applied Science, New Haven, CT 06511, USA

**Keywords:** PET imaging, GBM, biomarkers, Sigma 1, Sigma 2, PD-L1, PARP, IDH

## Abstract

Glioblastoma multiforme (GBM) is the deadliest type of brain tumor, affecting approximately three in 100,000 adults annually. Positron emission tomography (PET) imaging provides an important non-invasive method of measuring biochemically specific targets at GBM lesions. These powerful data can characterize tumors, predict treatment effectiveness, and monitor treatment. This review will discuss the PET imaging agents that have already been evaluated in GBM patients so far, and new imaging targets with promise for future use. Previously used PET imaging agents include the tracers for markers of proliferation ([^11^C]methionine; [^18^F]fluoro-ethyl-L-tyrosine, [^18^F]Fluorodopa, [^18^F]fluoro-thymidine, and [^18^F]clofarabine), hypoxia sensing ([^18^F]FMISO, [^18^F]FET-NIM, [^18^F]EF5, [^18^F]HX4, and [^64^Cu]ATSM), and ligands for inflammation. As cancer therapeutics evolve toward personalized medicine and therapies centered on tumor biomarkers, the development of complimentary selective PET agents can dramatically enhance these efforts. Newer biomarkers for GBM PET imaging are discussed, with some already in use for PET imaging other cancers and neurological disorders. These targets include Sigma 1, Sigma 2, programmed death ligand 1, poly-ADP-ribose polymerase, and isocitrate dehydrogenase. For GBM, these imaging agents come with additional considerations such as blood–brain barrier penetration, quantitative modeling approaches, and nonspecific binding.

## 1. Introduction

Glioblastoma Multiforme (GBM) is a fast growing, invasive brain tumor that typically results in death in the first 15 months after diagnosis [1]. It develops from glial cells, astrocytes or oligodendrocytes, and can evolve from lower-grade tumors or de novo. Previously, GBM was characterized as ‘grade IV’ astrocytoma. Recently, the World Health Organization (WHO) updated the classification of brain tumors to include genotypic markers, building on the histological markers considered previously [2]. Glioblastoma can be classified by a single nucleotide polymorphism in the isocitrate dehydrogenase (IDH) gene as wild-type or mutant. Approximately 10% of glioblastomas are IDH-mutant [2]. IDH-mutant status weakly predicts long-term survival (over 3 years post diagnosis) [3]. GBM tumors are heterogenous in location (with 25%–43% incidence in frontal lobes), histopathology, and the tumor microenvironment [4]. The first line of treatment for GBM is surgery, followed by radiation and chemotherapy [1]. Temozolomide, a DNA alkylating agent is often used for chemotherapy. In 2015, the vascular endothelial growth factor inhibitor Bevacizumab was fast-tracked for use in GBM after demonstrating efficacy in shrinking or halting tumor growth. However, it has failed to show improvement in overall survival [5]. Patients with GBMs have a very low survival rate with very few treatment options, making this a particularly acute health challenge. 

Medical imaging provides critical information for diagnosing, staging, and monitoring the treatment of GBM. While formal diagnosis relies on histopathology and genetic markers for grading, structural magnetic resonance images (MRIs) are routinely acquired and can be used in guiding surgery. Additional structural MRI methods can accurately classify and grade tumors with high accuracy, though it has not been adopted yet as common practice [6]. Positron emission tomography (PET) imaging provides important complementary information to anatomical MRI data. In this functional type of imaging, biochemical information about the tumor and the tissue surrounding it can be measured non-invasively. GBMs typically are fast growing, giving an important role for specific PET radioligands to quantify proliferation. PET imaging is also uniquely positioned to identify ideal cases for targeted treatments and evaluate treatment progression.

This article provides an overview of the novel imaging tracers used in PET imaging of brain tumors. Discussion includes the strengths, limitations, and pitfalls of individual imaging biomarker strategies, and general challenges associated with PET imaging of brain tumors. We first provide a brief overview of established PET imaging biomarkers (glycolysis, amino acid metabolism, DNA replication, hypoxia, and inflammation), followed by newer imaging targets (Sigma 1/ 2, programmed death ligand 1, poly-ADP-ribose polymerase, and isocitrate dehydrogenase) with promise to image glioblastoma lesions. None of these biomarkers are unique to glioblastoma, though their presence has been found in resected brain tumors. This work concludes with important quantitative considerations for use of these imaging biomarkers in the evaluation and treatment of GBM patients.

## 2. Overview of PET Imaging Agents for Brain Tumor

### 2.1. Sustained Proliferation Markers: Glycolysis, Amino Acid Transportation, and DNA Replication

The classic approach to imaging tumors in general, and in application to GBM, has been to probe the functional necessities of proliferation. These necessities include glucose metabolism, protein synthesis, and DNA replication. From a biochemical prospective, these functions highlight the ‘building block’ small molecules that compose macromolecules: sugars, nucleotide bases, and amino acids. 

**Figure 1:**
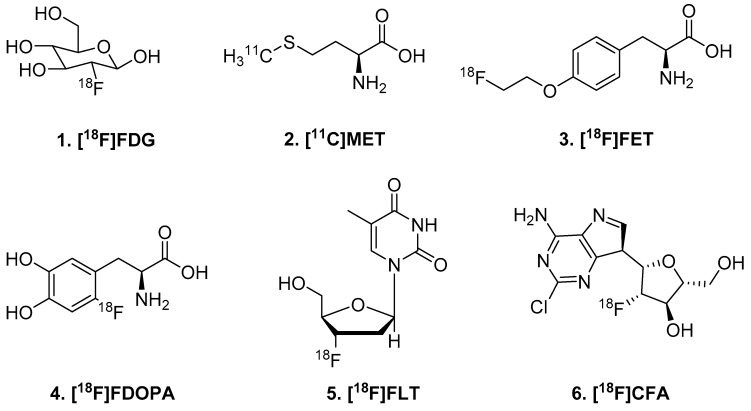
Radiotracers for markers of cellular proliferation.

Radionuclide-labeled forms of these building blocks have been employed to study these functions with PET imaging. The gold standard of most cancer imaging is [^18^F]FDG (**1**), a fluorine-18 glucose analogue. This radiotracer is actively taken up by the glucose transporter and participates in the first step of glucose metabolism (phosphorylation), then becomes trapped in the cell [7]. [^18^F]FDG PET allows for the functional imaging of glucose metabolism, a relative gold mine of information in most cancers. However, the brain has naturally high uptake of [^18^F]FDG, which complicates interpretation of GBM lesions near gray matter. Further efforts to image proliferation through the ‘building block’ strategy include neutral amino acid analogues ([^11^C]methionine (**2**); [^18^F]fluoro-ethyl-L-tyrosine (**3**); [^18^F]Fluorodopa (**4**)) and deoxynucleoside bases ([^18^F]fluoro-thymidine (**5**); and [^18^F]clofarabine (**6**)) (Figure 1). 

[^11^C]Methionine ([^11^C]MET) was developed shortly after [^18^F]FDG [8,9]; it was considered a valuable tracer because methionine, an essential amino acid, can be used in protein synthesis. Both L-and D-isomers were synthesized, and no difference in accumulation was observed [10]. This lack of selectivity between stereoisomers indicated that [^11^C]MET was not being incorporated for protein synthesis, as only L-amino acids are incorporated into proteins. Despite this, [^11^C]MET was still used as an alternative to [^18^F]FDG PET imaging because it is more sensitive (see review [11]). [^18^F]fluoroethyl-tyrosine ([^18^F]FET) was developed as another amino acid based alternative to [^18^F]FDG [12]. Unlike [^11^C]MET, [^18^F]FET is a modified amino acid, which led to questions about the radiotracer’s ability to be taken up into cells. In cellular experiments and tumor-bearing mice, it was found that [^18^F]FET is actively transported into cells [13]. The transporter responsible was later identified as l-amino acid transporter (LAT-1) [14]. [^18^F]FET PET combined with MRI can dramatically improve glioma identification and tumor diagnosis [15]. In terms of the diagnostic performance, MRI alone yielded a 96% sensitivity and 53% specificity; the combined technique achieved 93% sensitivity and 94% specificity [15]. While MRI is the gold standard for diagnosis and tumor staging, [^18^F]FET PET imaging provides complementary information in the form of increased specificity. 

The nonessential amino acid and neurotransmitter dopamine has also been used as a PET tracer in the form of [^18^F]L-fluoro-dihydroxyphenylalanine ([^18^F]FDOPA), which becomes a dopamine analogue in vivo after decarboxylation. [^18^F]Fluoro-dopamine can then be further taken up into vesicles by VMAT, or metabolized by monoamine oxidases or catechol-*O*-methyltransferase. This gives [^18^F]FDOPA a particular utility in neuroendocrine tumors [16] in addition to the obvious neurological application. [^18^F]FDOPA imaging in pre-operative glioma patients has shown a significant correlation between WHO grade and the volume of MRI contrast enhancement, volume of T2 hyperintensity, and [^18^F]FDOPA uptake (as SUV_max_ ratio of tumor to normal tissue) [17]. From this 45 patient cohort, a multivariate Cox regression suggested that [^18^F]FDOPA PET and age were significant prognostic factors for overall survival [17]. A major limitation of [^18^F]FDOPA use is the radiosynthesis. One of the first described high yielding radiosynthesis involved electrophilic fluorination [18]. Electrophilic fluorination is not a desirable method for routine clinical production because of the hazardous nature of F_2_ gas, however, improved syntheses were recently published involving nucleophilic fluorination [19]. 

Concurrent with the use of amino acid analogues, the deoxynucleoside base derivative [^18^F]fluorothymidine ([^18^F]FLT) was developed. [^18^F]FLT is trapped in tissues after phosphorylation by thymidine kinase [20]. This trapping was thought to be due to accumulation into growing DNA chains, as fluorothymidine was originally developed to terminate DNA, and used in HIV therapy. In reality, less than 1% of [^18^F]FLT was found to be incorporated in DNA in cellular studies, though it correlated highly with [^3^H]thymidine uptake [21]. Despite this lack of accumulation in DNA, [^18^F]FLT imaging confirms cellular uptake and correlates to Ki-67 expression on the corresponding resected tumor tissue [22]. Thymidine kinase is a principal enzyme in the DNA salvage pathway, and is most active in G1 and S phases of the cell cycle [23]. Because of its lower uptake in normal brain than [^18^F]FDG, glioma patients underwent [^18^F]FLT imaging and a similar correlation was observed with proliferative tissue markers [24]. When [^18^F]FLT PET was used to monitor treatment in malignant glioma trials and compared to MRI responses, [^18^F]FLT-PET was more predictive of overall survival than MRI [25]. Despite this utility, [^18^F]FLT is less widely used because of nonspecific binding in individuals [26], and because blood–brain barrier (BBB) penetration is limited, which limits its use specifically for glioma imaging.

An alternative method to [^18^F]FLT, which is phosphorylated by thymidine kinase, is to target deoxycytidine kinase (dCK). The activity of dCK, TK, and the other deoxyribonucleoside kinases are to provide an alternative pathway to de novo synthesis of DNA precursors [27]. Additionally, the activity of these kinases is critical for the activation of nucleoside analogues that are used for chemotherapy, like clofarabine [28]. Clofarabine is a chemotherapy used in pediatric patients with relapsed or refectory acute lymphoblastic leukemia. The radiolabeled [^18^F]clofarabine ([^18^F]CFA) has been evaluated in healthy humans and found to be BBB permeable [29]. Biodistribution of [^18^F]CFA showed uptake in lymph nodes, consistent with known dCK activity being required for T- and B-cell development [30]. In two patients with recurrent GBM, PET imaging with MRI was able to delineate specific regions of immune activity [31]. In one individual, the post-treatment PET-MRI scan demonstrated a 300% increase in immune cells in the tumor microenvironment, with the tumor volume remaining consistent. This combined technique has been shown in this preliminary data set to be useful in differentiating tumor progression from immune cell infiltration in a treatment monitoring scenario.

PET imaging of these classic markers of proliferation is useful to image GBM due to their simplistic design. However, changes in glucose metabolism, DNA replication, protein synthesis, and neurotransmitter homeostasis are not unique to cancers; they can describe a number of disease states. This generality makes these imaging approaches most appropriate for disease monitoring in cancer treatments like chemotherapy and targeted radiation. For further discussion of these tracers and their prognostic value in brain tumors, see a recent systematic review [32].

### 2.2. Hypoxia-Sensing Tracers 

The first-line treatment strategy for gliomas is surgical resection if possible, followed by chemotherapy and targeted radiation [33]. Chemotherapy and radiation are therapies that hinge on cell death by damaging DNA and initiating apoptosis. However, some tumors are resistant to these strategies. For example, it has been long known that poorly oxygenated tissue, or hypoxic tissue, is less sensitive to radiation. Gray et al. confirmed in 1953 that X ray therapy was more effective in mice that were breathing oxygen at a higher pressures than normal atmosphere [34]. Hypoxia has become a recognized key feature of most solid tumors [35]. In a hypoxic tumor microenvironment, radiation therapy could be more effective at a higher dose; however, this requires an accurate identification of that cell population. 

**Figure 2:**
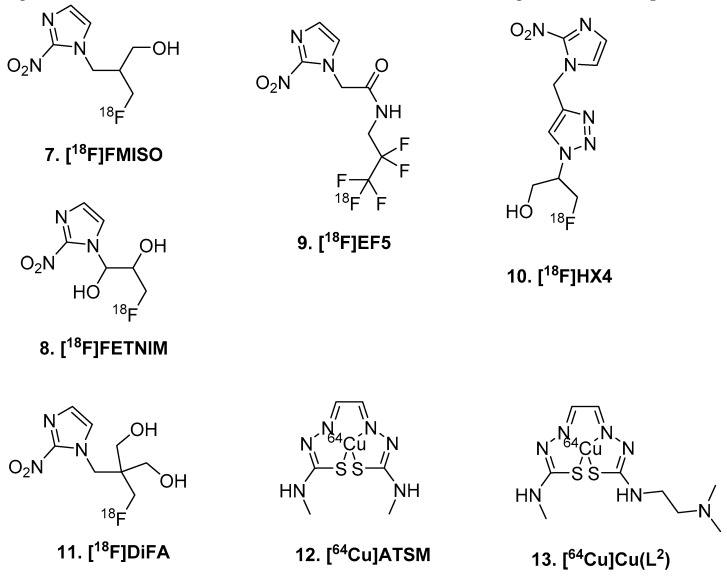
Hypoxia-sensing tracers.

PET radiotracers that sense oxygen levels in cells can be used to visualize hypoxia (Figure 2). [^18^F]Fluoromisoinodazole ([^18^F]FMISO; **7**) contains a nitroimidazole which is reduced to RNO_2_ radical after entering a viable cell. In the presence of oxygen, it will be re-oxidized and diffuse from the cell. If the cell is hypoxic, however, the radiotracer will be trapped. This tracer was first evaluated in V-79 cells using low O_2_ levels in the incubation to mimic hypoxia [36] and followed up with cancer models in animals [37]. Glioma patients have undergone [^18^F]FMISO PET imaging, though with limited success [38]. Chakhoyan et al. were able to build ptO_2_ maps from [^18^F]FMISO PET images and compare them to perfusion weighted imaging (MRI) and 1H-MR mono-voxel magnetic resonance spectroscopy (MRS) [39]. The correspondence with MRI and MRS imaging confirms the direct relationship between [^18^F]FMISO and oxygen levels in tissue. However, Valk et al. observed retention of [^18^F]FMISO in a GBM subject and anaplastic astrocytoma subject, but no retention of [^18^F]FMISO in another GBM subject [38]. [^18^F]FMISO PET imaging has a limited range in sensitivity between normoxic and hypoxic tissue [40]. Additionally, BBB penetration is low for [^18^F]FMISO which does not make it attractive for glioma imaging.

There are multiple hypoxia radiotracers developed around the same time as [^18^F]FMISO including [^18^F]FET-NIM (**8**), [^18^F]EF5 (**9**), and [^18^F]HX4 (**10**) (see review [35]). All of these ligands use the nitroimidazole moiety to sense oxygen level in vivo, and the differences in the chemical structures are primarily focused on the linker groups and location of fluorine-18 label (Figure 2). [^18^F]EF5 exhibits greater cell membrane permeability, slower clearance, and improved tumor uptake [41,42,43,44]. Its structurally very similar to [^18^F]FMISO and [^18^F]FETNIM, but incorporates a pentafluoro ethyl group via an amide linker. Labeling at this position requires electrophilic fluorination in the radiosynthesis, limiting regular production of this radiotracer. [^18^F]FETNIM was developed concurrently with [^18^F]FMISO, and is the most structurally similar with the addition of a hydroxyl group alpha to the nitroimidazole ring [45,46,47]. [^18^F]HX4 still contains the nitroimidazole, though added a triazole linker between this and the fluorine-18 label [48,49,50,51]. When compared to [^18^F]FMISO, [^18^F]HX4 provides the higher image contrast 4 h post-injection, but has high variability [52,53]. A second generation version of [^18^F]FMISO, [^18^F]DiFA (**12**) is slightly less lipophilic. This radiotracer aims to have a faster clearance rate, and thus improved signal-to-noise ratio over [^18^F]FMISO [54]. 

An alternative to [^18^F]FMISO, and the other organic oxygen sensing compounds, is [^64^Cu]ATSM (**12**), which uses a metal to sense oxidation changes (Cu(II) to Cu(I)) [55]. In comparison to [^18^F]FMISO, it has faster tracer kinetics and can reveal ‘hypoxic’ tissues in 15 min post injection. [^64^Cu]ATSM uptake is influenced not only by hypoxia, but also cellular concentrations of reducing species such as NADH [56]. Using [^64^Cu]ATSM imaging in glioma patients, SUV was found to be an independent predictor of both progression free survival and overall survival in this study [57]. In the GBM subgroup analysis, however, max SUV only showed significant prediction of progression free survival [57], using copper-64 allowed for synergy between PET and MRI, in this proof of concept using a closely related [^64^Cu]ATSM compound ([^64^Cu]Cu(L_2_); **13**), which has only been evaluated so far in vitro [58,59]. Though, it is likely to have similar BBB penetration issues as [^64^Cu]ATSM.

Hypoxia imaging techniques are important for the treatment planning of solid tumors to ensure effective regression. While current therapies do not target the hypoxia machinery in cells, this biochemical process remains a critical consideration for radiotherapy. Many tumor types, including gliomas, are described as being hypoxic and utilize PET or MR imaging methods for treatment planning [60]. This method also serves to monitor treatment after targeted radiotherapies. 

### 2.3. Inflammation

An important part of the immune response is inflammation and, like peripheral cancers, brain cancer cells will trigger this response. A histological analysis of lesions from 1265 patients with glioblastoma multiforme identified the presence of lymphocytes and reactive astrocytes [61]. Inflammation has been explored as a therapeutic target for such cases. Nonsteroidal anti-inflammatory drugs (NSAIDs) have been evaluated to suppress the growth of tumor cells in vitro [62]. While NSAIDs are an inappropriate therapy for GBM because of the lack of targeting, inflammation is a characteristic of GBM pathology and could be useful for imaging.

**Figure 3:**
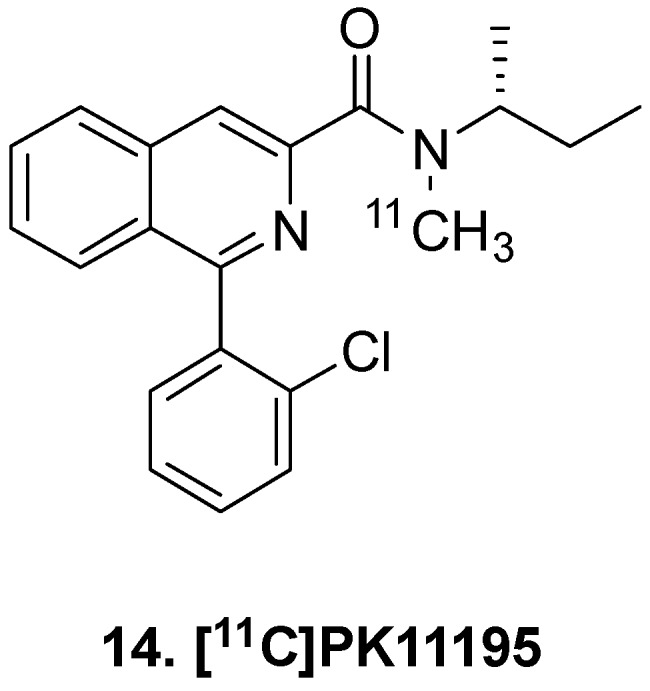
TSPO ligand.

Translocator protein (18 kDa), TSPO, is an integral mitochondrial membrane protein responsible for cholesterol transport and responds to cell stress. Broadly, TSPO is treated as a biomarker sensitive to pro-inflammatory stimuli, and small molecule inhibitors of TSPO have been utilized in PET imaging [63]. [^11^C]PK11195 (**14,** Figure 3) was developed for general use of inflammation imaging, and even in glioma patients [64]. Immunohistochemical experiments with patient tissue further confirmed increase TSPO mRNA and protein levels [65]. Furthermore TSPO PET imaging was correlated to outcome [66]. In a larger prospective trial which included tumor biopsy, imaging and histology were found to correlate, and *BP*_ND_ in high-grade gliomas was significantly higher than in low-grade astrocytomas and low-grade oligodendrogliomas [67]. Although second and third generation TSPO radioligands with higher specific binding have since been developed, TSPO imaging in GBM has major limitations including tumor heterogeneity and inability to distinguish signal caused by radiation therapy from signal due to the tumor microenvironment. 

## 3. New Biomarkers for GBM PET Imaging

New strategies for cancer therapeutics target proliferation (sigma 2), immunity (sigma 1, PD-L1), and genetic modification (PARP, IDH). PET imaging agents have developed in tandem with advancement of these therapies. These therapies were initially designed to treat more prevalent, peripheral tumors. The nature of heterogeneity in brain tumors inspires the use of biomarker specific imaging agents, as opposed to the more general ligands for proliferation, hypoxia, and inflammation. Applications to brain cancers comes with the significant considerations of blood–brain barrier penetration and effectiveness. The PET imaging agents discussed in the following section are not yet utilized in human brain imaging but represent promising candidates for the new therapeutic and/or biomarker strategies. 

### 3.1. Sigma 1

A key characteristic of GBMs is their invasiveness, which leads to the very low survival rate. Sigma 1 and sigma 2 receptors are expressed in the human tumor cell lines: C6 glioma, NIE-115 neuroblastoma, and NG108-15 neuroblastoma- glioma hybrid [68]. While sigma 1 is associated with invasiveness, sigma 2 receptors have been associated with proliferation. Both receptors are interesting PET imaging targets for glioblastoma. 

**Figure 4:**
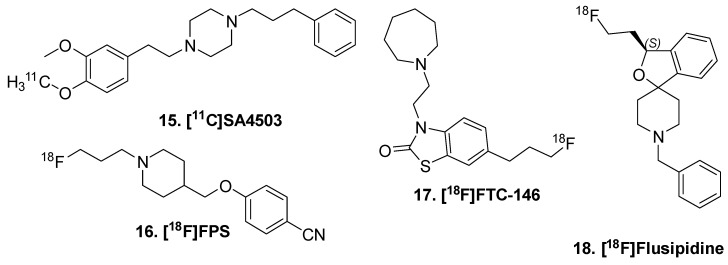
Sigma 1 receptor radioligands.

Sigma 1 receptor (S1R) was identified as the site of action of the antipsychotic haloperidol [69]. Its role in the CNS has been investigated for neurodegenerative disorders [70] in addition to GBM. The first PET ligand for S1R was [^11^C]1-(3,4-dimethoxyphenethyl)-4-(3-phenylpropyl)piperazine ([^11^C]SA4503: **15,** Figure 4) [71]. While it showed nanomolar affinity to S1R, it unfortunately has high affinities for other receptors, ion channels, and second messenger systems [72]. Both S1R and S2R have structural similarity to opiate receptors, which necessitates specificity and selectivity when designing ligands. Additionally, in the development of fluorine-18 sigma-1 receptor ligands, very high binding affinity has corresponded to very slow clearance rate. For example, [^18^F]1-3-fluoropropyl-4-((4-cyanophenoxy)-methyl)piperidine ([^18^F]FPS; **16**) [73] did not reach pseudo-equilibrium by 4 h in human [74]. Another ligand, [^18^F]6-(3-fluoropropyl)-3-(2-(azepan-1-yl)ethyl)benzo[d]thiazol-2(3H)-one ([^18^F]FTC-146; **17**) was also found to be irreversible and not suitable for neuroimaging [75]. Recently, [^18^F](S)-Fluspidine (**18**) [76] was developed and evaluated in human [77]. The pharmacokinetics are improved, and with xenograft mouse models of glioblastoma have visible increases in radioligand binding. 

### 3.2. Sigma 2

The amino acid and nucleoside base PET imaging approach is a classic, straightforward way to assess functional proliferation. The sigma-2 receptor (S2R; TMEM97) has recently been implicated in cancer biology; S2R levels increased 5-fold in proliferating tumor cells compared to quiescent tumor cells [78]. This allows for radioligand design for a specific protein more akin to drug design. For an in-depth history of S2R ligand development, see references [79,80].

**Figure 5:**
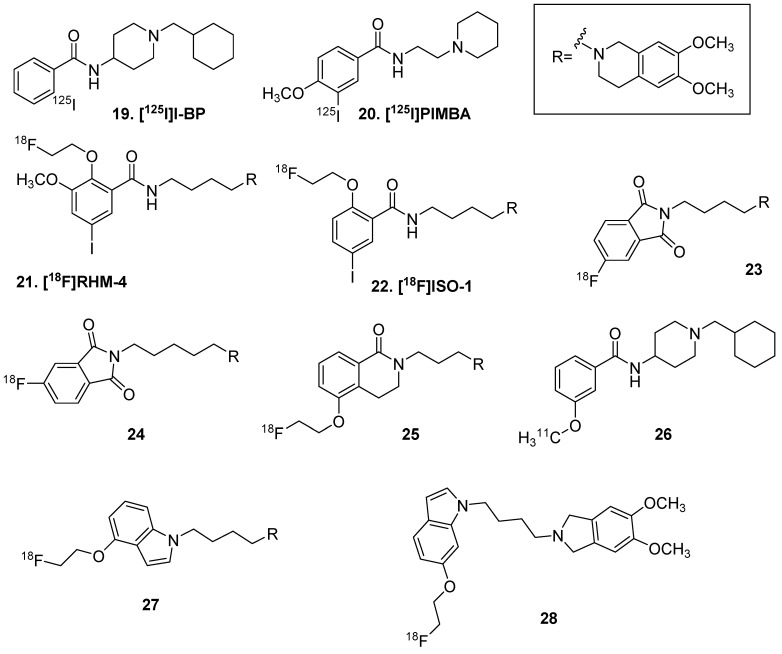
Sigma 2 receptor radioligands.

An early imaging effort for S2R was using 4-[^125^I]BP (**19**, Figure 5), a small molecule with high affinity for both sigma 1 and 2 receptors [81]. Further efforts by these authors led to [^125^I]PIMBA (**20**), though this radioligand suffered from high background binding [82]. [^18^F]RHM-4 (**21**) was developed and demonstrated S2R overexpression in pancreatic cancer, though BBB penetration was not investigated [83]. The radioligand [^18^F]ISO (**22**) was also developed, structurally similar to **21** except lacking the aryl methoxy group [84]. A positive correlation is observed between **22** binding and tumor Ki67 expression [85]. In treatment monitoring of CDK4/6 inhibition plus endocrine therapy in breast cancer xenograft animals, **22** was found to assess more delayed changes related to cell cycle arrest compared to [^18^F]FLT [86]. However, **22** is not taken up into the brain, based on organ residence studies in rodent [85]. This has inspired alternative ligands with BBB penetration as the design goal.

A scaffold incorporating pthalimides (**23** and **24**) showed elevated brain uptake and specific binding (displaceable with cold ligand and haloperidol), though the tumor to background ratio was low [87]. Abate et al. described **25**, which demonstrated good in vitro binding and specificity; however, as a P-glycoprotein (PGP) substrate it is not suitable for brain imaging [88]. The same group continued with a carbon-11 effort (**26**), based on the inhibitor PB28 and very similar to **19**; however, brain uptake was low and the compound did not display high enough specific binding [89]. Wang et al. reported two fluorine-18 inhibitors, **27** and **28**, with high brain uptake in mice in 2017, though it may be a PGP substrate as well [90]. These efforts have demonstrated some challenges in the radioligand design of sigma 2 receptor inhibitors for brain imaging, i.e., PGP efflux and limited specific binding. 

### 3.3. PD-L1

Avoiding destruction by immune cells is a powerful strategy utilized by cancer cells. Until recently, imaging research in this space has focused on antibody-based strategies, which is challenging due to limited brain penetration. The molecules used in PET imaging for Programmed death ligand 1 (PD-L1) are large molecule therapeutics, including antibodies, antibody fragments, and peptides. An early imaging effort developed at Johns Hopkins University adapted the therapeutic antibody for PET imaging: [^64^Cu]Azetozolizumab [91]. The same group later developed a peptide, [^64^Cu]WL12, although brain was not listed in the biodistribution study [92]. Another protein effort from the Gambhir lab at Stanford, [^64^Cu]NOTA-HACA-PD1 and a gallium 68 version, determined no brain uptake definitively [93]. Merck developed an affibody Z_PD-L1_1_ that was fluorine-18 labeled; however, brain penetration has not been demonstrated, though an affibody is more likely to be BBB penetrant than the preceding antibodies [94]. 

**Figure 6:**
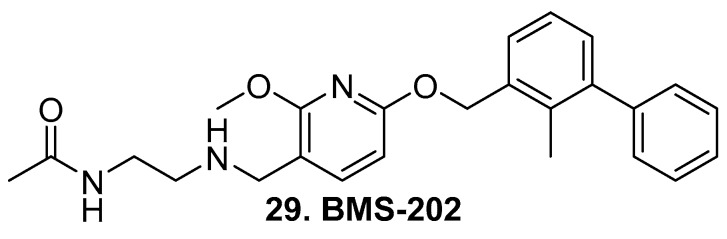
Small molecule PD-L1 inhibitor.

Although small molecule inhibitors have been in development, to date no small molecule radioligands have been described. Bristol Meyers Squib has the first small molecule inhibitor reported, BMS-202 (**29**, Figure 6) [95] and has characterized the binding in a crystal structure [96]. The mechanism of action is thought to be selectively induced dimerization of PD-L1, which inhibits binding to PD-1 (see reviews [97,98,99])

### 3.4. PARP

ADP-ribose polymerase (PARP) is the enzyme responsible for attaching linear or branched polymers of ADP onto broken DNA and other biomolecules. PARP-1 recognizes single and double-strand breaks, crossovers, cruciform, and supercoils. Additionally, PARP-1 maintains the stability of replication forks, and the basal activity is very low [100]. In cancer cells containing a BRCA2 deficiency, inhibiting PARP causes synthetic lethality [101]. This treatment strategy has been used to develop multiple small molecule therapeutics [102]. 

**Figure 7:**
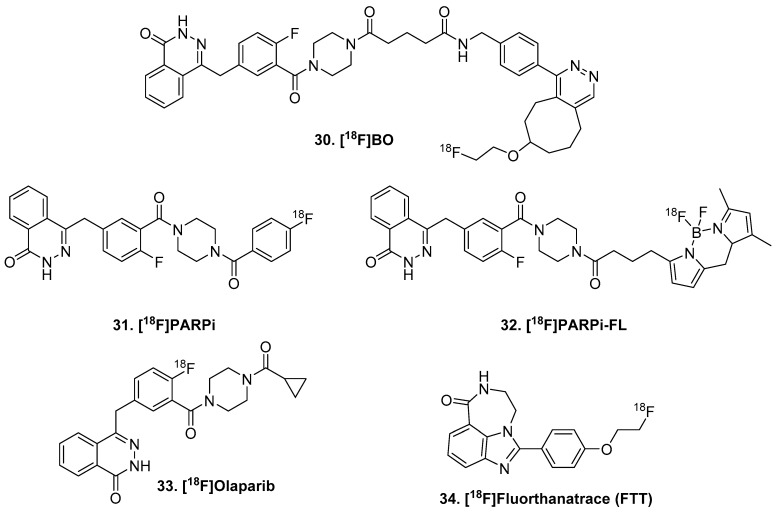
PARP ligands.

The first in-class inhibitor olaparib has been adapted for use in PET imaging: [^18^F]BO (biorthogonal olaparib; **30**, Figure 7) [103]. [^18^F]BO demonstrated ubiquitous distribution in cancer cells and localization within the nucleus in cancer cells [104]. Structurally similar to olaparib, the inhibitor radioligand [^18^F]PARPi (**31**), and corresponding fluorescent version (**32**), has been used in GBM cell lines [104] and in rodent [105]. Although this shows specific binding in peripheral tumors, there was no significant blocking with brain uptake at 2 h biodistribution. Brain penetration was very low initially. In comparison to [^11^C]choline and [^18^F]FLT, PARPi demonstrated a lower mean uptake in tumor than the other two PET ligands; however, the lower background uptake enabled PARPi to delineate brain tumors in rodent models with more clear contrast [106]. The Gouverneur group recently radiolabeled olaparib itself with fluorine-18, though limited imaging studies have been done with this tracer [107]. As expected, [^18^F]olaparib (**33**) was taken up selectively in PARP-1 expressing cells and in mouse tumors. Additionally, radioligand uptake was increased by 70% after tumor irradiation, indicated a great potential for monitoring radiation damage. However, brain was not included in biodistribution calculations and there does not appear to be brain uptake in the dynamic PET images. Unlike the preceding PARP radioligands, the inhibitor [^18^F]fluorthanatrace ([^18^F]FTT; **34**) is not based on olaparib, but rather rucaparib. This benzimidazole carboxamide derivative is highly potent with an IC_50_ of 6.3 nM [108]. It has been evaluated in humans, though with low brain penetration [109]. There remains a need for BBB penetrating small molecule inhibitors of PARP in order to be useful specifically in glioblastoma imaging. 

### 3.5. Isocitrate Dehydrogenase (IDH)

Through genome wide association studies (GWASs), the common mutation R132H, located on the isocitrate dehydrogenase (IDH) 1 gene, was found in more than 70% of grade II and III astrocytomas, oligodendrogliomas, and glioblastomas that developed from these lower-grade lesions [110]. However, only 10% of glioblastomas are IDH mutant [1], and this is weakly associated with tumor aggressiveness [3]. IDH- mutant GBM has a significantly longer survival rate compared to IDH wild type, 31 months compared to 15 months with standard treatment.

The observed IDH mutation essentially eliminates all enzymatic activity [111]. In normal cells, IDH1 converts isocitrate to α-ketoglutarate and form NADPH, which maintains a pool of reduced glutathione and peroxiredoxin. When isocitrate cannot be converted to α-ketoglutarate, it instead is converted to R(-)-2-hydroxyglutarate [112]. 2-Hyrdoxyglutarate and α-ketoglutarate are cofactors for many enzymes and their availability influences DNA methylation status. 2-HG inhibits 5mC hydroxylase (TET2) and lysine demethylases (KDM) leading to demethylation of DNA and histone, respectively. This changes gene expression and thus tumorigenesis. R-2HG stimulates EgIN1, which promotes HIF1a degradation by hydroxylation [113]. Therapeutic inhibitors are being developed for IDH1 to influence this pathway and similarly slow tumorigenesis and promote survival. 

Ivosidenib (AG-120) is the first in-class IDH1 reversible inhibitor [114]. In rats, some brain penetration has been demonstrated, 4.1% after 50 mg/kg dose, which could indicate some effectiveness in glioblastoma. The phase I trial in low grade glioma subjects is ongoing (NCT03343197). Another inhibitor (IDH305) for mutant IDH1 is in development by Novartis, with strong data for binding in brain homogenate [115]. In a phase I clinical trial, safety was evaluated in glioma, AML/MDS, and other/ non-CNS solid tumors with IDH mutation [116]. However, phase II trials of IDH305 in glioma has been withdrawn, possibly due to liver toxicity (NCT02977689 [117]).

**Figure 8:**
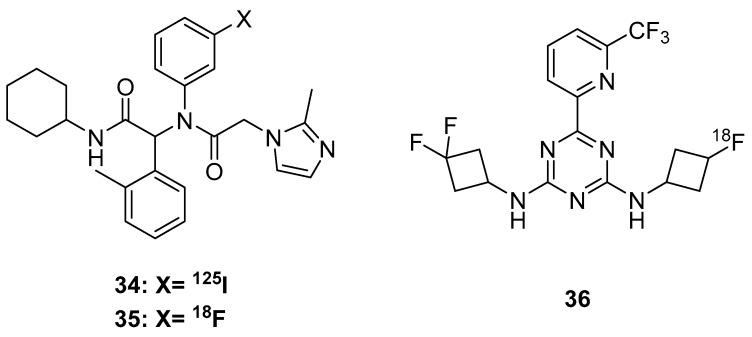
Isocitrate dehydrogenase (IDH) radioligands.

Before small molecule inhibitors of IDH were available, PET radioligands were evaluated for their relationship to IDH mutation status. While [^18^F]FDOPA does not correlate with IDH mutation status [118], it appears that [^18^F]FET imaging does significantly associate with IDH mutation status [119,120,121], although there does not appear to be a biochemical relationship between [^18^F]FET uptake and IDH mutation status. With the disclosure of small molecule IDH inhibitors came the preliminary development of IDH-selective PET radioligands. Chitneni et al. utilized an iodine-131 and fluorine-18 version (**35**, Figure 8) of AGI-5198; however, these compounds lacked selectivity for mutant IDH over wild type [122]. In a follow up from the same group, [^18^F]triazinediamine (**36**) analogues were radiosynthesized, based on Enasidenib (AG-221) [123], The K_d_ (**36**) was calculated to be 40 nM with a B_max_ of 4426 gmol/mg in a mutant anaplastic astrocytoma cell line, which is promising. However, biodistribution studies showed bone uptake from radiolytic defluorination, so further design is required [123].

## 4. General Imaging Considerations

Brain PET imaging for any disease state is challenging. The blood–brain barrier (BBB) poses a major obstacle for any successful radiotracer targeting glioblastoma. Even if the radiotracer enters the brain, many compounds exhibit slow brain entry (i.e., small *K*_1_ values). Since glioblastoma lesions often compromise BBB, increased radioactivity concentrations at the lesion site may reflect increased nonspecific radiotracer instead of, or in addition to, increased target signal. For example, a combined [^18^F]FMISO PET and MRI study noted high uptake of [^18^F]FMISO in areas of BBB disruption as well as in necrotic tissue [124]. This raises a challenge in quantification at a suspected glioblastoma lesion. Kinetic modeling approaches incorporating dynamic data, in some cases, may help distinguish nonspecific signal from specific signal in some cases. Although these scanning protocols can require longer scanning time and possibly arterial blood sampling, the potential for improved quantification of specific radiotracer uptake offers important benefits to evaluating diagnosis, staging, or treatment efficacy that must be considered when using PET to image GBM. 

Reference region approaches offer alternatives for quantitative analyses that may shorten the length of scanning time and do not require arterial blood sampling. While such approaches have major limitations in cases of ubiquitously expressed targets, such as TSPO, in the case of brain tumor imaging the reference region may be drawn as a larger region that is removed from the lesion. For example, in a case of [^18^F]FET imaging a fixed-size reference ROI was placed in hemispheres contralateral to tumorous tissue, which yielded fully image-derived measures that correlated with disease-free survival [125]. Image-derived input function allow for alternative non-invasive modeling approaches. For example, [^18^F]FMISO has no reference region, but image-derived tissue-to-blood ratios provide reasonable proxies for measured parent radioactivity in venous blood [126]. Such approaches can maintain quantitative accuracy while reducing logistical complexities introduced by full dynamic scans with blood sampling. 

A final major obstacle for PET radiotracers imaging GBM is high nonspecific binding. For example, [^18^F]FMISO, [^18^F]FLT, and [^64^Cu]ATSM exhibit elevated nonspecific binding, which limits their usefulness. Such radiotracers exhibit low signal to noise ratio (SNR), which makes it more difficult to differentiate areas of low uptake from noise. In contrast, radiotracers with low nonspecific binding may be amiable to reference region analysis if non-tumor brain regions exhibit negligible specific binding. Off-target binding poses another related challenge. For example, sigma 1 and sigma 2 receptors have significant structural similarities to opiate receptors. These challenges highlight the need for blocking studies with candidate radiotracers to confirm suitable sensitivity and specificity without high nonspecific binding. 

## 5. Conclusions

PET imaging provides important functional information about GBM tumors and surrounding tissue environment. Markers of proliferation, hypoxia, and inflammation have been used to image the lesions of GBM patients. Exciting new frontiers for PET imaging targets for GBM include PD-L1 for immune status, PARP-1 for DNA damage, sigma 2 receptors as alternative markers of proliferation, and isocitrate dehydrogenase for tumorigenesis activity. These PET imaging targets have the potential to enhance diagnosis, staging, and treatment approaches for GBM. As GBM PET imaging techniques advance, it is critical to consider blood brain barrier penetration and nonspecific binding in the evaluation of new radioligands.
